# Proportional Control with Pole-Placement-Tuned Gains for GPS-Based Waypoint Following, Experimentally Validated Against Classical Methods

**DOI:** 10.3390/s26010255

**Published:** 2025-12-31

**Authors:** Heonjong Yoo, Wanyoung Chung

**Affiliations:** Department of Electronic Engineering, Pukyong National University, Busan 48513, Republic of Korea; yuhj0819@pknu.ac.kr

**Keywords:** cornering indexing method, global positioning system point, application program-ming interface, trajectory tracking like goalpoint following, state flow block, vector definition

## Abstract

The paper focuses on the goal point following an algorithm design based on the exact Global Positioning System (GPS) points. In order to achieve that, the first GPS point and initial heading angle are previously calculated by recursively adopting GPS points from the Naver Application Programming Interface (API) map. The GPS points are designated as a goal point in order to follow the mobile platform to the generated path. Simulation and experimental results demonstrate that goal point following logic can be implemented based on the generated path achieved from the map. Furthermore, the goal-point-following method is extended to trajectory tracking by defining the vector rather than the designated goal point. The result is demonstrated through simulation and an experiment with the real mobile platform.

## 1. Introduction

The goal-point-following algorithm has been studied for years in [1]. Furthermore, the way point navigation method is introduced based on GPS data sets described in [2]. In such papers, there are Pure Pursuit algorithms that are used for following designated goal-points. In this presentation, we address a modified stanley goal-point-following problem by transforming it into trajectory tracking-like goal-point-following algorithm for which vector definition rather than designated goal-point definition is required. Our previous work used state-flow method [3] to develop a laptop-based experiment using the state flow algorithm provided by software MATLAB. The state flow method performs path tracking of the generated path and checks whether the Ranger mini mobile platform accurately tracks the generated path dynamics by inspecting observed longitude and latitude, heading angle information in real time. Furthermore, the state flow algorithm is revised for the goal point as briefly described in [4]. On the other side of the research, trajectory tracking methods for four-wheel mobile platforms have been researched in several articles. First of all, the sliding mode control and predictive control of the model is applied for the trajectory tracking in [5]. Secondly, assistance quality analysis and robust control is applied for the trajectory tracking of electric vehicle in [6]. Thirdly, a second-order sliding mode and a nonlinear disturbance observer technique are applied for the tracking of the electric vehicle trajectory in [7]. Lastly, an elaborated resistance network approach of artificial potential fields is applied for the tracking of the autonomous vehicle trajectory described in [8]. In recent years, Reinforcement Learning has been studied in various fields, particularly in path following problems on mobile platforms; see [9]. In particular, the deep deterministic policy gradient (DDPG) algorithm is applied for autonomous ground vehicles (AGVs) regarding the path-following subject in [10]. In addition to that, the reinforcement learning method is incorporated into the mobile robot simulator to show that the platform follows the designated waypoint in [11]. More recently, a 3D path-following control method was proposed using a linear active disturbance rejection control and DDPG algorithm, explained in [12]. Furthermore, the dynamical obstacle avoidance experiment for the mobile platform is implemented in [13].

Previously, we use the goal-point-following method rather than trajectory control, since the goal-point-following is more accurate at the end point of arrival. At this point, we extend the result into the trajectory-tracking-like goal-point-following method and propose the aforementioned result for accurate goal-point tracking and smooth trajectory tracking. Studies in [14,15,16,17,18] have extensively explored path-following methods for systems of this type. To summarize this, we make the following contribution in this paper.

- Despite the various aforementioned control and estimation methods, the experiment was conducted focusing on the waypoint-following logic incorporated into ROS publish and subscribe block introduced in MATLAB/SIMULINK recently.
- The real environment experiment is implemented based upon the waypoint from ROS subscribe block.
- Furthermore, the trajectory-tracking-like goal-point-following is implemented through simulation and the real mobile platform experiment by defining vectors rather than designated goal-points.

## 2. The Cornering Complicated Path Using Cornering Index

The complicated cornering path included is generated and experimented in the building of the Chungbuk National University.

**Figure 1:**
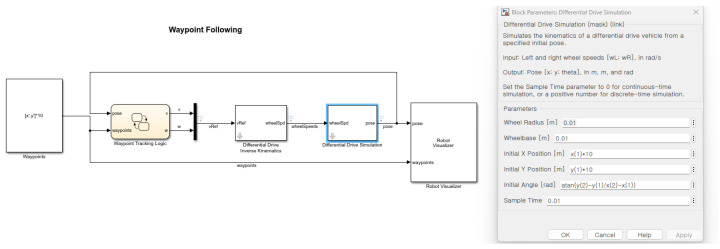
The specification setting in the Simulink (* means multiplcation sign).

In order to match the Robot operating system (ROS) odometry block data sets into the longitude and latitude of MATLAB 2022b Web map data sets, the specification setting in the Simulink is required. The setting of the block parameter requires the initial position and the initial heading angle represented by the following Figure 1.

**Figure 2:**
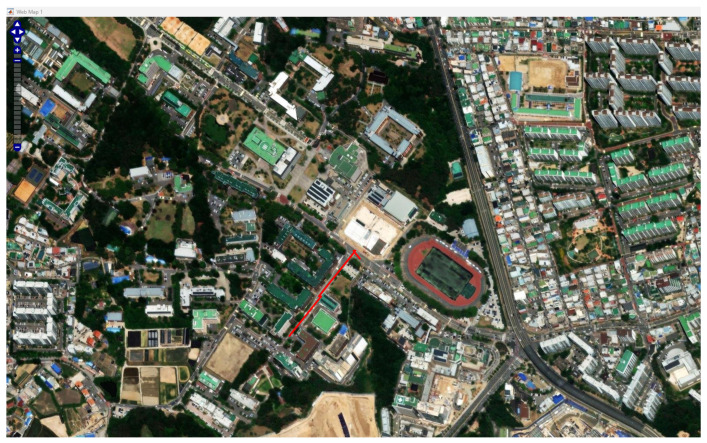
The generated path in MATLAB Web map.

The path generated on the MATLAB web map is represented by Figure 2 shows the reference path generated from MATLAB web map by reading the longitude and latitude information.

**Figure 3:**
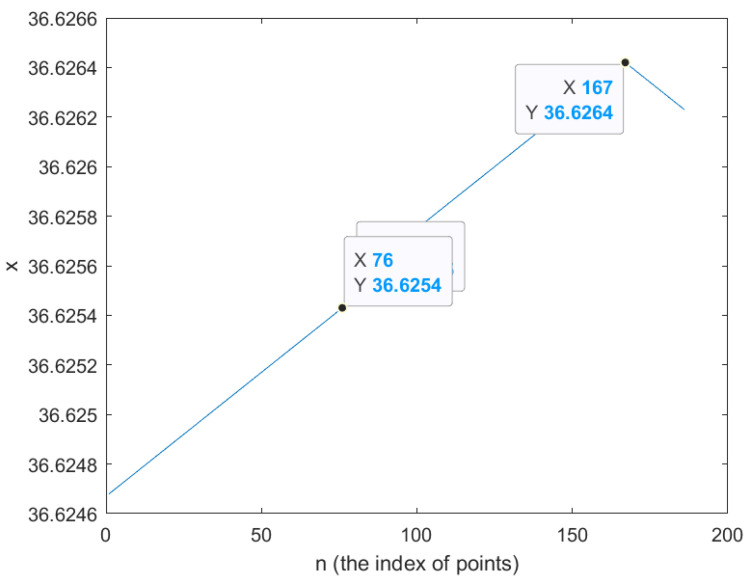
The longitude points to figure out the index of points in turning points.

**Figure 4:**
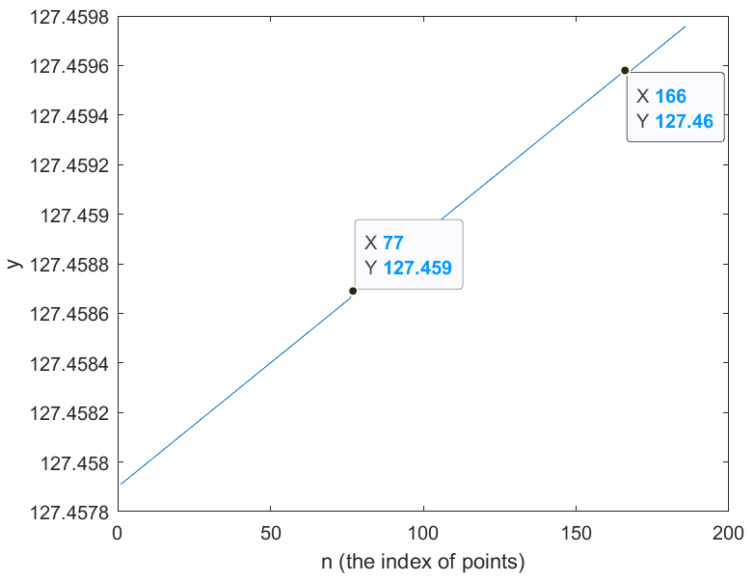
The latitude points to figure out the index of points in turning points.

The black lines have two cornering points and sequentially lead to the end point. The following Figure 3 and Figure 4 show that the index of turning points is approximately 76 and 166. Hence we set the mediate points as 76 and 166.

**Figure 5:**
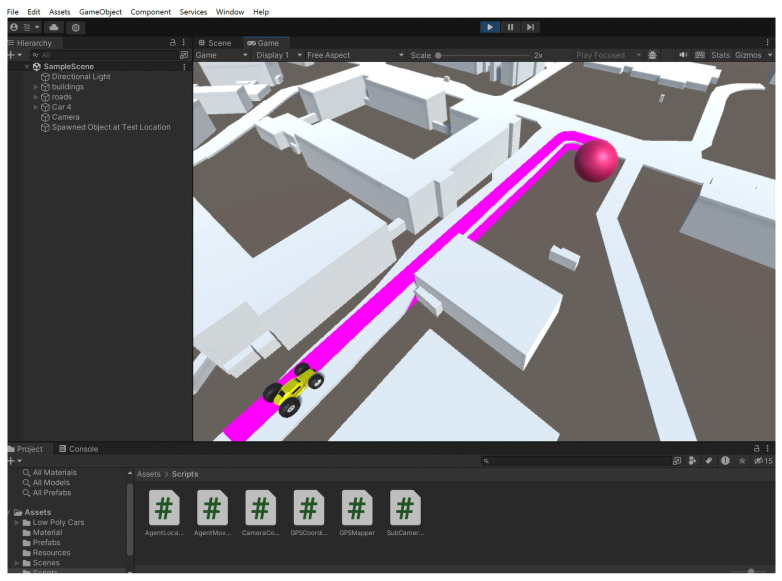
The GPS trajectory from unity program.

After the experiment, the following Figure 5 shows that the ROS odometry data sets are the same as the Unity GPS data. The slope inside Pukyong National University is the GPS trajectory colored pink. The 3D CAD is the building within Pukyong national university accepting Google site.

In Figure 3 and Figure 4, the index numbers of the turning point are approximately 76 and 166 by observing the turning line. Therefore, the setting parameters are given in Table 1.

In Table 1, the waypoint spacing is set to 6 m. This value was empirically chosen. The experiments were conducted at a constant speed of 0.2 m/s, which is significantly lower than the robot’s maximum speed of 2.6 m/s. While this lower speed ensures safe testing and minimizes dynamic effects, it should be noted that the tracking behavior may differ at higher speeds.

**Figure 6:**
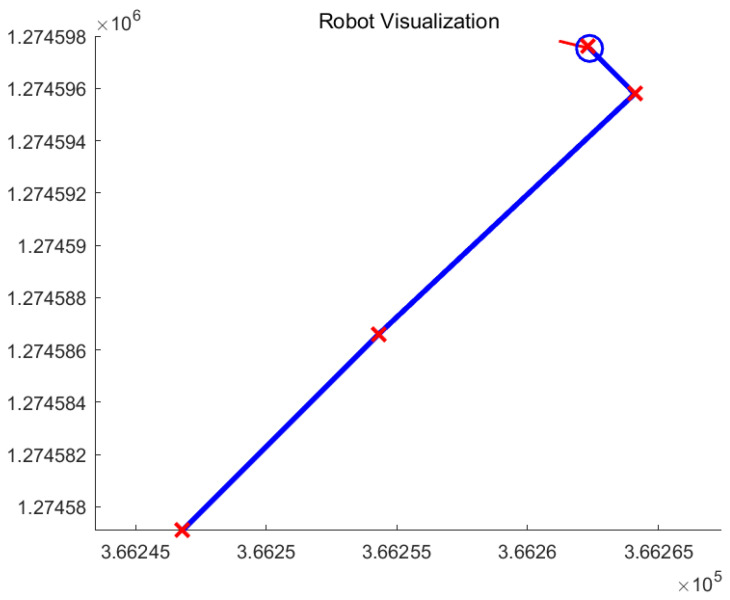
The actual path based on the indexing number of points using waypoint-following in MATLAB webmap.

Figure 6 shows that the blue line represents the trajectory followed by the robot and the red crosses represent the designated conering points.

The optimal trajectories are implemented through the configuration of the goal-points setting, $\left(x_{1},y_{1}\right),\left(x_{end},y_{end}\right)$ and the distance is 2 m, described in Table 1. In addition to that, the path that includes cornering was implemented using intermediate points $\left(x_{1},y_{1}\right)$, $\left(x_{76},y_{76}\right)$, $\left(x_{166},y_{166}\right)$, and $\left(x_{end},y_{end}\right)$; the index number is extracted through the longitude and latitude plot. Initially, the spatial profiles of the linear and angular velocities, denoted as $v_{i}$ and $\omega_{i}$, are computed along the planned trajectory based on the waypoint-following strategy. These velocity profiles are subsequently mapped to the temporal domain using the Simulink simulation environment, where each velocity value is associated with a corresponding time instant. Throughout the motion, the wheeled mobile robot operates with a linear velocity $v_{i}$ and an angular velocity $\omega_{i}$. In the next stage, the spatial velocity variables $v_{i}$ and $\omega_{i}$ are transformed into time-dependent signals $v_{t}$ and $\omega_{t}$. This transformation is carried out by discretizing the trajectory into a sequence of short time intervals $P_{i}$, which are defined according to the sampling period of Simulink. Within each interval between $P_{i}$ and $P_{i+1}$, the linear and angular velocities are assumed to remain constant due to the sufficiently small sampling time. The experimental process here is described in [3]. A key difference from [3] is that, while the experiments in [3] were conducted by measuring time and distance without using GPS points, in this work we generate velocity profiles based on the exact target GPS points until the robot reaches its destination. This approach allows for a trajectory-following experiment directly referenced to GPS coordinates, distinguishing our method from that of [3].

## 3. Automatic Detection of Cornering Points Using Directional Change Analysis

To improve scalability and generalizability, cornering points on the reference path are automatically detected by analyzing directional changes between consecutive points, instead of manual identification. Let the reference path be represented as a sequence of discrete points:

(1) $$P=\{\left(x_{1},y_{1}\right),\left(x_{2},y_{2}\right),\ldots ,\left(x_{n},y_{n}\right)\}.$$

For each intermediate point i∈{2,…,n−1}, define the two vectors:

(2) $$v_{1}=\left(x_{i}-x_{i-1},\;y_{i}-y_{i-1}\right),$$

(3) $$v_{2}=\left(x_{i+1}-x_{i},\;y_{i+1}-y_{i}\right).$$

The cornering point $\theta_{i}$ at point *i* is computed as the angle between $v_{1}$ and $v_{2}$:

(4) $$\theta_{i}=\arccos\left(\frac{v_{1}\cdot v_{2}}{∥v_{1}∥\;∥v_{2}∥}\right),$$

where · denotes the dot product and ∥·∥ is the Euclidean norm. A point *i* is classified as a cornering point if its cornering point exceeds a threshold θth:

(5) $$i\in \mathrm{Cornering}\;\mathrm{points}\;⟺\;\theta_{i}>\theta \mathrm{th}.$$

**Figure 7:**
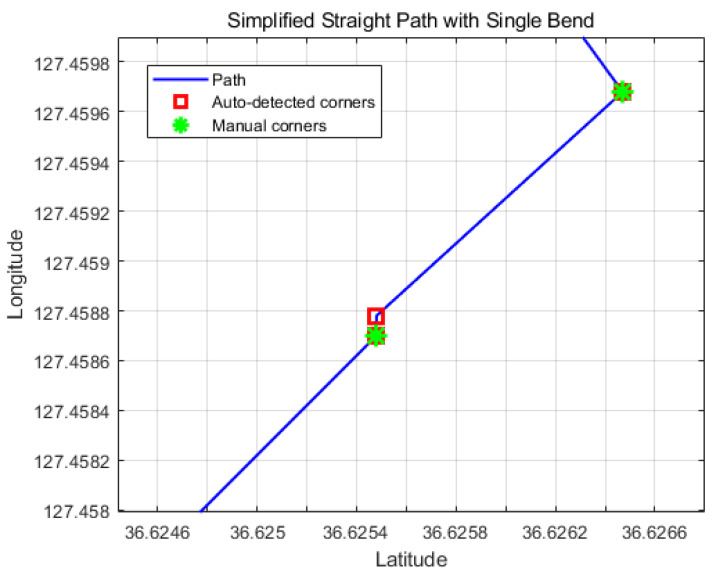
The comparison of automatic detection corner indices with manual detection corner indices.

This automated approach enables objective extraction of turning points, enhancing the method’s applicability to various paths without manual intervention. The threshold angle θth can be tuned according to path complexity and sensitivity requirements. This method replaces manual waypoint indexing, improving reproducibility and scalability. A point *i* is classified as a cornering point, and its corresponding index *i* is recorded as a cornering index, if its directional change exceeds a threshold θth. We implemented automatic detection code, and compare the manual detection given as Figure 7.

Since the experimental data sets were unavailable during the review, we generated a random path and applied both manual and automatic corner detection. The comparison indicates that the automatically detected corners perfectly match the manually identified ones. In this study, the threshold angle for corner detection, θth, was set to $30^{\circ }$ (approximately 0.524 rad). This value was chosen to detect prominent turning points while ignoring minor deviations along the path.

### Sensitivity Analysis of Threshold Angle

**Figure 8:**
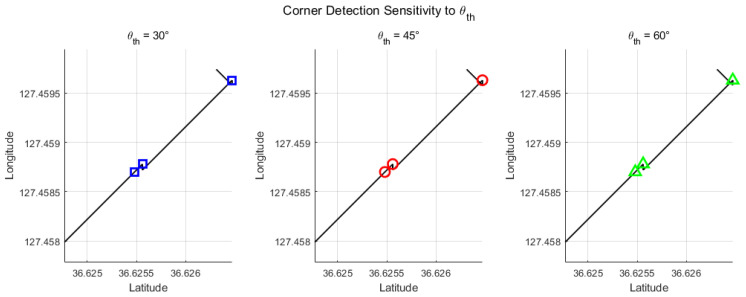
Automatic corner detection results for different threshold angles: $30^{\circ }$ (blue squares), $45^{\circ }$ (red circles), and $60^{\circ }$ (green triangles).

To evaluate the impact of the threshold θth on corner detection, we tested three different values: $30^{\circ }$, $45^{\circ }$, and $60^{\circ }$. Figure 8 shows the detected corner points for each case.

As observed, all tested values of θth ($30^{\circ }$, $45^{\circ }$, and $60^{\circ }$) result in the same corner detections for this relatively simple path. This indicates that the method is robust to the choice of θth under such conditions, although more complex paths may require tuning to adjust sensitivity.

## 4. Exact GPS Point Extraction

In this section, the exact GPS point is extracted using the NAVER API (Application Programming Interface). By reading the GPS points from the text file, the vector definition in the MATLAB prompt is done by designating the longitude information as *X* data sets and the latitude information as *y* data sets. Although the implementation in this study utilizes the NAVER Maps API, which is specific to Korea, the proposed framework is fundamentally API-agnostic. The method relies on standard map features such as waypoint retrieval, routing, and coordinate transformation, all of which are commonly supported by major global map services including Google Maps, OpenStreetMap, and Mapbox. Therefore, with appropriate interface adjustments, the framework can be readily adapted to other map service providers without loss of functionality.

### Design of Goal-Point Follower Based on GPS Point

**Figure 9:**
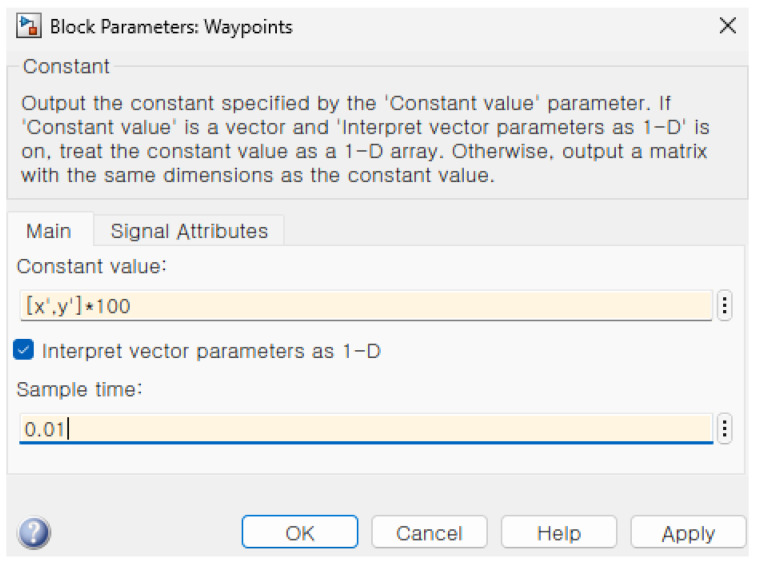
The definition of the vector specification (* means multiplication sign).

In the previous section, each point was entered as a goal point, but in this section, a path-following method of waypoint following was used by inserting all points as goal points. In Figure 9, the setting is a whole vector rather than each point of the goal point. In this manner, the achievement of the trajectory tracking using the waypoint is implemented through experiment. By implementing the aforementioned method, smooth tracking is possible for the trajectory tracking.

The mobile robotics simulator adopts GPS points as a goal point, then follows the designated goal point, which is the same as the GPS point from the NAVER API (Application Programming Interface).

**Figure 10:**
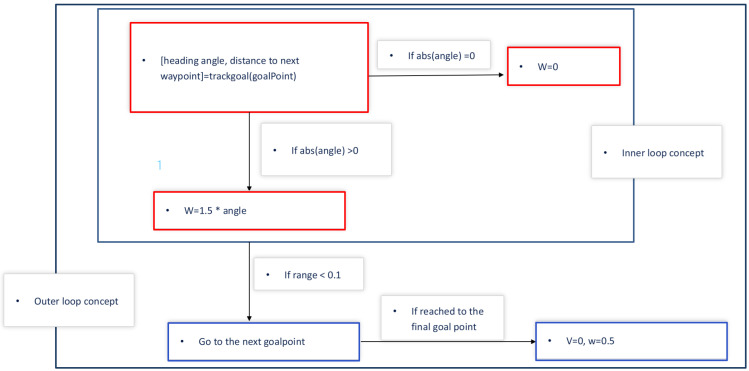
Goal-point-following design in the state flow block (* means multiplication sign).

The goal-point-following design in the state flow block is implemented in MATLAB/Simulink. There is an inner loop in which the feedback gain 1.5 is calculated from the pole placement design in [14]. The method is described in Section 4. This means that the current position converges exponentially to the goal point with the exponential component of 1.5. In addition to that, there is a outer loop design in which the next goal point is found and goes to the next goal point until the range is below 0.1. Here, range means the distance between the current position and the target position. The purpose of the simulator generates the linear and angular velocities signal that moves the mobile platform of the four wheels based on GPS data sets. The whole process is described in Figure 10.

**Figure 11:**
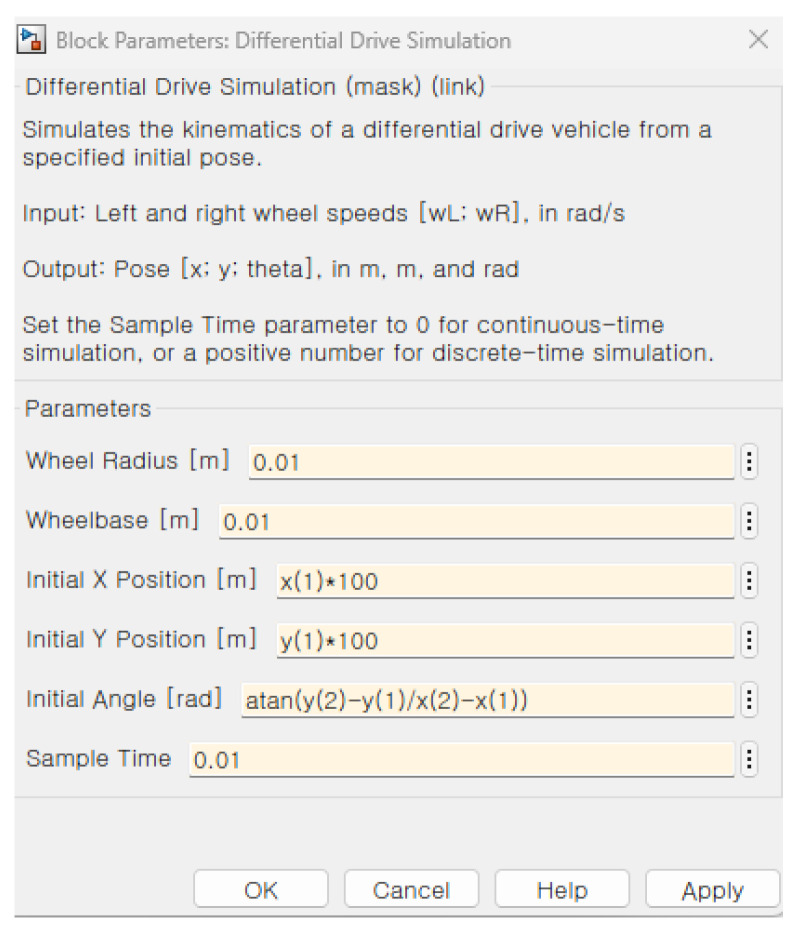
The specification of the initial point and initial heading angle (* means multiplication sign).

Figure 11 shows the block parameter in the mobile robotic simulator. The initial longitude and latitude information and the heading angle are calculated on the basis of the GPS point from the NAVER API in Figure 11.

Figure 9 shows the setting of the block parameter 1. The explanation of the block parameter is about the constant block in Simulink explained in Korean in Figure 9. The crucial point here is that we set the vector definition $\left[x^{\prime },y^{\prime }\right]$ rather than the designated goal point described in Table 1.

## 5. Pole Placement Method for Feedback Gain 1.5 in the Goal-Point-Following Design

(6) $$\begin{array}{c} u-[v;w],y=[x,y,h]\\ sys=tfest(u,y,np)\\ b=[01138019.49];a=[13235055.32];\\ \left[A,B,C,D]=tf2ss(b,a\right) \end{array}$$

*v* and *w* are the linear and angular velocities input; on the other hand, *x*, *y*, and *h* are longitude, latitude, and heading angle information. From that, transfer estimation tfest in MATLAB is used to find the system matrices A,B,C,D, also described in [15].

Now, the linear system modeling is given as

(7) $$\begin{array}{c} \dot{x}\left(t\right)=Ax\left(t\right)+Bu\left(t\right)\\ y(t)=Cx(t) \end{array}$$

The controller gain K=1.5 is found by the pole placement given as

(8) $$\begin{array}{c} \lambda \left(A-BK\right)=\lambda \left(\left[\begin{array}{cc} -0.0010 & 0\\ 1 & 0 \end{array}\right]-\left[\begin{array}{c} 1\\ 0 \end{array}\right]\left[\begin{array}{cc} 1.5 & 1.5 \end{array}\right]\right)\\ =(-0.7505+0.9679\times j,\;-0.7505-0.9679\times j)\\ K=\left[\begin{array}{cc} 1.5 & 1.5 \end{array}\right] \end{array}$$

If the system modeling displays a two-time scale system, the pole placement technique can be extended into the two-time scale design in [16]. At this point, the gain *K* is utilized for the proportional controller gain design in the state flow design described in Figure 10.

## 6. Application of the Four-Wheel Mobile Platform Results Using the Aforementioned Method

### 6.1. Experimental Condition

The experiments in this paper were conducted using the Ranger Mini Version 2 mobile platform. The physical specifications and motion limits of the robot are summarized as follows:

- **Dimensions (L × W × H):** 738 mm × 500 mm × 338 mm, providing a compact footprint suitable for indoor and outdoor environments.
- **Wheelbase:** 494 mm; **Axle track:** 364 mm.
- **Maximum Speed:** up to 2.6 m/s, which defines the upper limit of the platform’s linear motion in path-following experiments.
- **Drive Configuration:** Four-wheel drive with independent steering (4 WD).
- **Weight:** Approximately 135 kg with a single battery; capable of supporting additional payloads depending on configuration.

**Figure 12:**
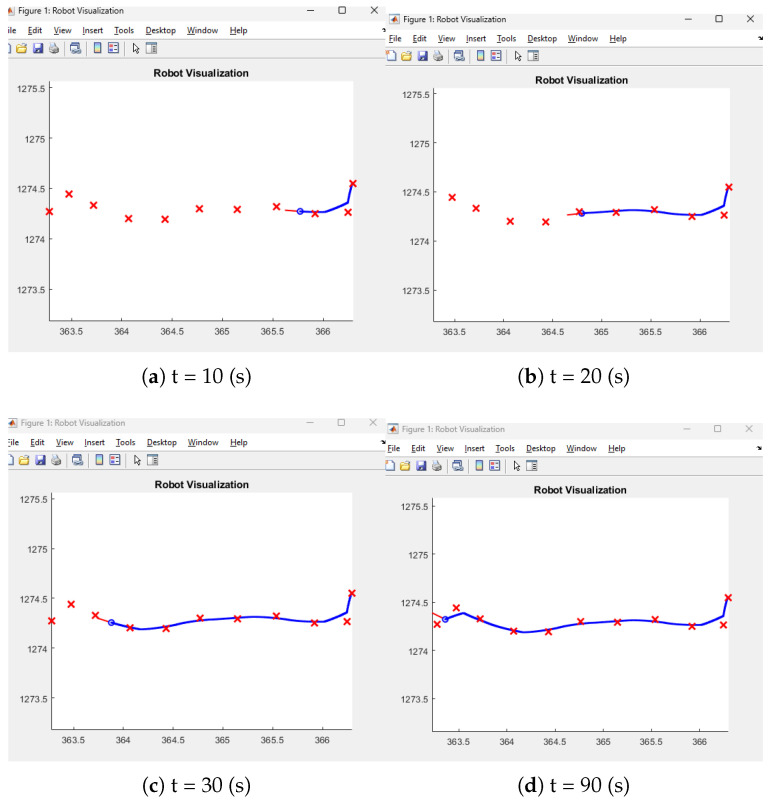
The screenshot for the goal-point-following simulator based on exact GPS points.

**Figure 13:**
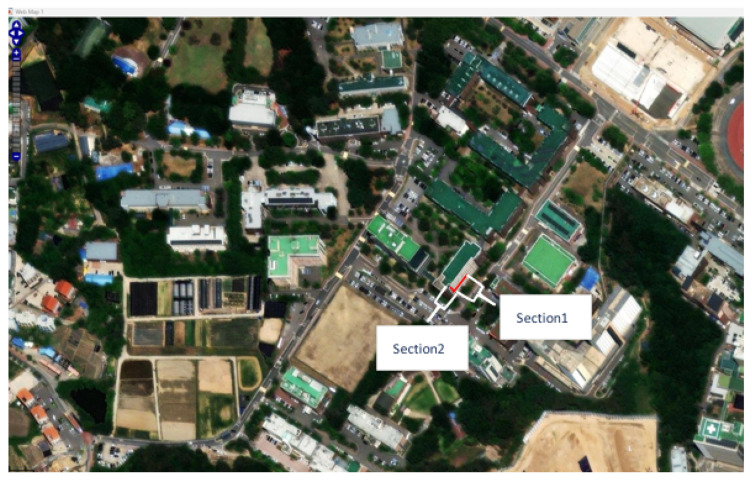
The extraction of the longitude and latitude points within MATLAB web map.

All experiments were conducted using the TDR-3000 RTK-GPS receiver, TDR-3000, Wego robot, Seoul, Republic of korea, which provides centimeter-level positioning accuracy under optimal conditions. Manufacturer specifications indicate a horizontal accuracy of approximately ±3 cm (RMS) with 97% confidence in open-sky environments. The following Figure 12 shows that the state-flow range-following mechanism is inserted the robot program mechanism in the MATLAB/Simulink. Firstly, the whole point of the indoor environment is extracted by moving the mobile platform based on the designated line. Then the vector definition on the MATLAB prompt is implemented by typing $\left[x^{\prime },y^{\prime }\right]$. Figure 13 shows how to extract the odometry longitude and latitude information within the Web map.

Section 1 is a straight line; section 2 is the straight line on the right in Figure 13. After that, the definition in MATLAB is performed by typing $\left[x^{\prime },y^{\prime }\right]$ on the prompt. For a concise description of the path following the result, the vector definition is required instead of using the designated way point. In this manner, trajectory-following-like way points are possible, in which the mobile platform can be moved smoothly and precisely.

We compare the actual coordinates and GPS point; this is successfully matched in Figure 13.

### 6.2. Design and Methodology

**Figure 14:**
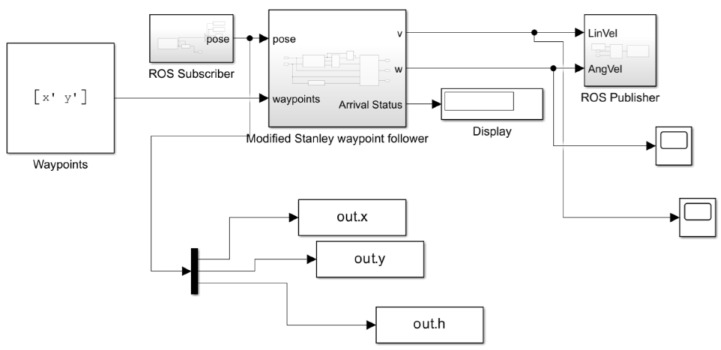
The overall MATLAB/Simulink model.

**Figure 15:**
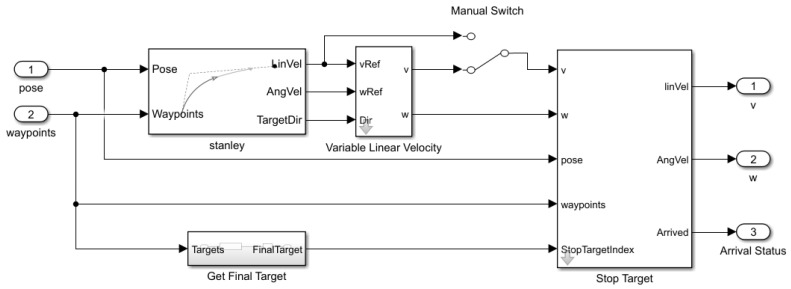
The subsystem modeling for stanley goal-point-following design.

**Figure 16:**
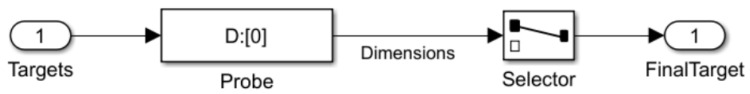
The subsystem modeling for the final target design.

Figure 14 shows the overall MATLAB/Simulink structure during the real mobile platform’s experiment. At this point, we set the vector definition $\left[x^{\prime },y^{\prime }\right]$ rather than designated goal point $\left(x_{1},y_{1}\right),\left(x_{76},y_{76}\right),\left(x_{166},y_{166}\right),\left(x_{end},y_{end}\right)$ in Section 2. Figure 15 and Figure 16 are subsystems of Figure 14. Using the “Rosinit” command in MATLAB, the following Simulink structure is connected into the four-wheel mobile platform through the Ubuntu Laptop, Samsung, Seoul, Republic of korea.

Figure 15 and Figure 16 are subsystem designs for the overall MATLAB/Simulink model.

**Figure 17:**
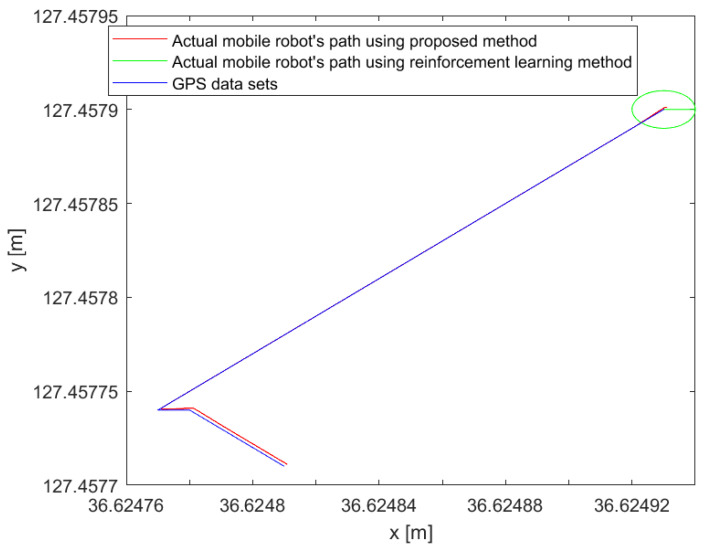
The comparison of the proposed method with the existing reinforcing method in [11].

**Figure 18:**
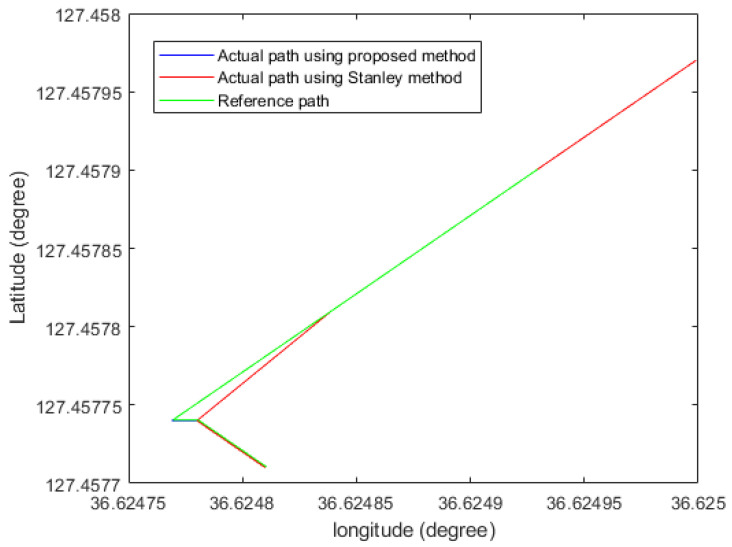
The comparison of the proposed method with the Stanley method.

**Figure 19:**
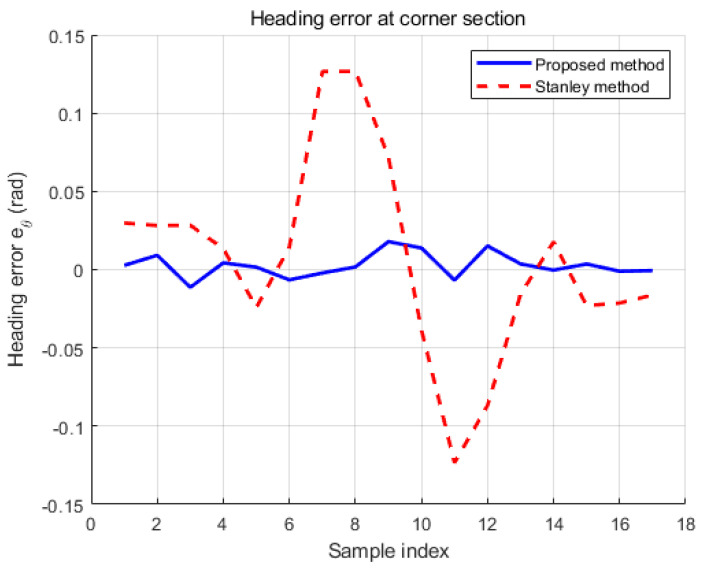
Comparison of heading errors in cornering sections between the proposed method and the Stanley method.

In Figure 17, the four-wheel mobile platform tracks the highly accurate GPS points based on Table 2. The root mean square error (RMSE) between the generated reference path based on Table 2 and the experimental result is 3 cm, which is an improvement of 4.5 cm over the conventional method given in [17]. Table 3 compares the original Lyapunov method for RMSE. The state flow method in [3] shows smooth trajectory tracking, but the root mean square error (RMSE) at the turning point is 8 cm, which is larger than the proposed method. Furthermore, the reinforcement learning method proposed by artificial intelligence shows that it is in place from the starting point, since it requires learning time given the potential points. However, the proposed method does not show holding time from the starting point, which is beneficial compared to the artificial intelligence (AI) method. In addition to that, Figure 17 shows the comparison of the proposed method with the existing reinforcement learning method. The reinforcement learning method required time to collect data sets. Before acquiring data, the autonomous vehicle moved to another point and took a detour to the next designated point described in the green line in Figure 17. On the other hand, the proposed method displays that the mobile four-wheel platform could directly go to the designated goal point without hesitation. In the figure, the green line is the platform’s. The green line represents the trajectory obtained using the reinforcement learning approach, where the mobile platform exhibits circular motions and hesitation due to the required learning phase. In contrast, the blue line shows the trajectory using the proposed goal-point-based method, in which the platform directly converges to the labeled points without hesitation. The red line denotes the generated reference path. In Figure 18, the proposed method is compared with the classical Stanley method. The proposed approach demonstrates superior tracking performance, particularly in cornering sections. In contrast, the classical Stanley method exhibits larger deviations when following GPS points in cornering regions. Figure 19 shows the time histories of the heading error $e_{\theta }$ at a representative cornering section during the experiments. Although both controllers generate similar spatial paths, a clear difference is observed in their heading behaviors. The proposed method maintains a heading error close to zero throughout the cornering maneuver, indicating smooth and anticipatory alignment with the reference direction. In contrast, the Stanley controller exhibits noticeable oscillations and larger heading error deviations at the corner, which result from repeated corrective steering actions when negotiating the turn. These results highlight that the proposed framework improves heading regulation at cornering sections, even when the overall path tracking performance appears comparable in the Cartesian space.

For a fair comparison, the gains of the Stanley and Pure Pursuit controllers were tuned using the same experimental dataset and performance criteria as the proposed method. All controllers were tuned to minimize the RMS lateral and heading tracking errors.

The GPS measurements used in this study are subject to inherent positioning errors caused by satellite geometry, atmospheric conditions, and sensor noise. In order to quantify the GPS accuracy, a static experiment was conducted at a fixed location for 10 min. The collected longitude and latitude data were converted into local Cartesian coordinates. The positioning error is evaluated using the root mean square error (RMSE), defined as

(9) $$\mathrm{RMSE}=\sqrt{\frac{1}{N}\sum_{i=1}^{N}\left({\left(x_{i}-\bar{x}\right)}^{2}+{\left(y_{i}-\bar{y}\right)}^{2}\right)},$$

where $\left(x_{i},y_{i}\right)$ denotes the measured GPS position and $\left(\bar{x},\bar{y}\right)$ is the mean position. The experimental results indicate that the GPS error remains within ±0.3 cm for 97% of the measurements. This error is modeled as a bounded disturbance acting on the position states. Since the proposed controller is designed based on state flow and pole placement technique, the closed-loop system remains input-to-state stable (ISS) under bounded GPS disturbances.

### 6.3. Clarification of the Proposed Trajectory-Tracking-like Goal-Point-Following

Although the proposed method utilizes a vector form of reference points, its contribution is not merely the interpolation of waypoints. The key distinction from classical waypoint-based approaches such as Stanley or pure pursuit lies in the control structure and reference update mechanism. In conventional waypoint-following methods, the controller tracks discrete target points and switches to the next waypoint once a distance threshold is satisfied. This discrete switching often causes transient oscillations or hesitation behavior, especially near cornering regions. In contrast, the proposed method represents the entire GPS waypoint sequence as a continuous reference vector, allowing the controller to smoothly follow the path in a trajectory-like manner. By incorporating the waypoints in vector form, the method effectively enables continuous, corner-aware waypoint progression, rather than simply interpolating between isolated goal points. Moreover, unlike Stanley or pure pursuit controllers, which are primarily geometric and heuristic in nature, the proposed approach is formulated as a nonlinear feedback control problem. The error dynamics are explicitly defined, and controller gains are selected based on pole placement and Lyapunov stability analysis. As a result, the method guarantees asymptotic convergence of the tracking error, while preserving the practical advantage of accurate goal-point arrival. Therefore, the proposed approach can be interpreted as a bridge between classical goal-point-following and trajectory tracking, emphasizing smooth, vector-based waypoint progression rather than conventional discrete waypoint switching.

## 7. Computational Requirements and Runtime Performance

The proposed trajectory-tracking-like goal-point-following method is designed to operate in real time with low computational overhead. At each control cycle, the algorithm performs basic vector operations, trigonometric evaluations, and proportional feedback calculations. No online optimization, matrix inversion, or iterative learning process is involved.

### 7.1. Computational Complexity

The computational complexity per control step is O(1), as the control inputs are computed directly from the current state and the next reference waypoint. This is comparable to classical geometric controllers such as Pure Pursuit and the Stanley method.

### 7.2. Runtime Performance

The controller was implemented in MATLAB/Simulink with Stateflow and executed at a fixed sampling time of 0.01 s (100 Hz). In all simulation and real-world experiments, the computation time per control cycle remained well below the sampling interval, ensuring real-time execution without deadline violations.

### 7.3. Comparison with Standard Methods

In terms of computational demand, the proposed method has similar runtime characteristics to the Pure Pursuit and Stanley controllers, as summarized below:

- **Pure Pursuit:** Requires geometric look-ahead calculations and curvature estimation.
- **Stanley Method:** Computes cross-track error and heading error using trigonometric operations.
- **Proposed Method:** Computes pose error in the robot frame and applies linear feedback control.

While the computational cost is comparable across these methods, the proposed approach achieves improved tracking accuracy in cornering sections without increasing computational complexity. This makes it suitable for embedded and real-time robotic platforms.

## 8. Applicability and Limitations

The proposed GPS-based waypoint-following method relies on continuous and reliable GPS signals. While it performs well in open and structured environments, its performance may degrade in areas where GPS reception is poor or intermittent. Such environments include urban canyons, indoor spaces, or dense forests, where signal dropout and multipath effects can significantly affect positioning accuracy.

### 8.1. Suitable Environments

- Open areas such as parks or campuses;
- Structured outdoor environments with clear GPS reception.

### 8.2. Unsuitable Environments

- Urban canyons with tall buildings;
- Indoor environments;
- Dense forests or heavily obstructed areas.

## 9. Discussion

In the presentation, the vector definition instead of the designated target point is applied to the four-wheel mobile platform. Using the aforementioned design, smooth trajectory tracking and exact goal point tracking is possible, in which both benefits are adopted. Previously, the state flow method is applied in [3]; however, the four-wheel mobile platform could not recognize the current longitude and latitude information based on the state flow method. In the proposed method, the mobile wheel platform four could recognize the current position based on GPS data sets, then the benefit of smooth trajectory tracking and exact direction of the destination point is achieved. We would like to clarify that quantitative performance metrics were indeed used in the experimental evaluation. Specifically, the tracking performance was quantitatively assessed using the root mean square error (RMSE) between the reference GPS trajectory and the experimentally measured path. These quantitative results are reported in the revised manuscript and demonstrate a clear improvement over the baseline methods. Regarding the experimental environment, the test scenario was intentionally designed to be simple and static. The primary objective of this study is to validate the effectiveness of the proposed goal-point-following strategy and its trajectory-tracking-like behavior based on precise GPS points. Since obstacle avoidance is not directly related to the proof of concept of goal-point convergence and smooth trajectory tracking, it is beyond the scope of the present experimental validation. We emphasize that the proposed framework can be naturally extended to environments with obstacles by integrating higher-level planning or obstacle avoidance modules, which will be addressed in future work. Furthermore, the proposed trajectory-tracking-like goal-point-following method demonstrates high accuracy in both simulation and real-world experiments; several practical limitations should be noted:

- **GPS signal degradation:** The current implementation relies on accurate GPS measurements. In urban environments, GPS dropouts or multipath effects can degrade the positioning accuracy, potentially reducing the performance of the path-following controller. Future work will investigate sensor fusion techniques, such as integrating IMU and wheel odometry, to improve robustness against GPS outages.
- **High-speed operation:** The mobile platform is modeled as a unicycle-like system, which assumes low-to-moderate speeds. At higher velocities, dynamic effects such as wheel slip, inertia, and actuator limits may violate these assumptions. Extending the control design to account for dynamic models is a potential future direction.
- **Environmental complexity:** The experiments conducted were limited to structured environments with pre-defined paths. Deploying the method in more complex or cluttered environments will require additional obstacle avoidance strategies and robustness analysis.

Furthermore, all experiments were conducted on a single campus, which may limit the generalizability of the results. The novelty of this work does not lie in the introduction of a new control paradigm, nor in the use of Stateflow or ROS themselves, but in the engineering formulation, tuning, and experimental realization of a trajectory-tracking-like goal-point-following framework for GPS-based mobile robots. The main contributions of this paper are summarized as follows:

- A **trajectory-tracking-like goal-point-following formulation** is proposed, which bridges conventional goal-point-following and continuous trajectory tracking by defining the reference as a vector of GPS points rather than discrete target points.
- A **systematic error dynamics and feedback control law** are derived for the proposed formulation, enabling exponential convergence of position and heading errors with analytically selected gains.
- An **automated cornering point detection method** based on cornering point analysis is introduced, replacing manual waypoint indexing and improving scalability and reproducibility.

## 10. Conclusions

The point inside Pukyong national university was extracted via Unity program, then compared to the actual data sets from the generated reference path in the MATLAB web map. The conering indexing method was introduced in the paper to precisely control it. Secondly, the goal-point-following in the state flow block design is introduced, and the pole placement technique is applied to the controller gain. By applying the aforementioned method, the goal-point-following based on the exact GPS point is implemented in MATLAB/Simulink. When it is implemented, we use the vector definition which is the trajectory-tracking-like goal-point-following method. Lastly, the aforementioned vector control rather than designated goal-point-following method is applied to the real mobile platform trajectory tracking experiment by adopting the GPS points inside Pukyong National University. We also demonstrated that the latitude *x*, longitude *y*, and direction angle *h* follow the shortest path between the buildings of the Pukyong National University.

## 11. Related Work

The method of following the point has been previously studied in [4]. The reinforcement learning method is applied to the method described in [11]. The proposed method is compared with the reinforcement method and the existing waypoint-following method in Figure 17.

## Figures and Tables

**Table 1:** Experiment condition outdoor environment.

	Initial Points	Way Points
Point with index number	$\left(x_{1},y_{1}\right)$	$\left(x_{1},y_{1}\right),\left(x_{76},y_{76}\right),\left(x_{166},y_{166}\right),\left(x_{end},y_{end}\right)$
Other condition	Distance 6 m	Sampling time setting: 0.01

**Table 2:** Longitude, latitude odometry data sets.

Place Dissection	Longitude x(t)	Latitude y(t)
Section 1	$\left[\begin{array}{c} 36.624929999999999\\ 36.624919999999996\\ 36.624910000000000\\ 36.624899999999997\\ 36.624890000000001\\ 36.624879999999997\\ 36.624870000000001\\ 36.624859999999998\\ 36.624849999999995\\ 36.624839999999999\\ 36.624829999999996\\ 36.624820000000000\\ 36.624809999999997\\ 36.624800000000000\\ 36.624789999999997\\ 36.624780000000001\\ 36.624769999999998 \end{array}\right]$	$10^{2}\times \left[\begin{array}{c} 1.274579000000000\\ 1.274578900000000\\ 1.274578800000000\\ 1.274578700000000\\ 1.274578600000000\\ 1.274578500000000\\ 1.274578400000000\\ 1.274578300000000\\ 1.274578200000000\\ 1.274578100000000\\ 1.274578000000000\\ 1.274577900000000\\ 1.274577800000000\\ 1.274577700000000\\ 1.274577600000000\\ 1.274577500000000\\ 1.274577400000000 \end{array}\right]$
Section 2	$\left[\begin{array}{c} 36.624780000000001\\ 36.624790000000004\\ 36.624800000000000\\ 36.624809999999997 \end{array}\right]$	$10^{2}\times \left[\begin{array}{c} 1.274577400000000\\ 1.274577300000000\\ 1.274577200000000\\ 1.274577100000000 \end{array}\right]$

The data sets are collected from MATLAB web map regarding longitude and latitude information of Section 1 and 2.

**Table 3:** RMSE comparison for different path-following methods and sensitivity analysis on the gain *K* for the proposed method.

Method/Gain	RMSE [cm]	Notes
Proposed method (K=1.0)	3.7	Slightly lower gain
Proposed method (K=1.5)	3.0	Default, analytically derived
Proposed method (K=2.0)	3.5	Slightly higher gain
Previous work (Ref. [17])	7.75	Baseline reference
Pure Pursuit	14.3	Standard path-following controller
Stanley	12.8	Standard path-following controller

## Data Availability

The original contributions presented in this study are included in the article. Further inquiries can be directed to the corresponding author.

## Nomenclature

GPS	Global positioning system
API	Application programming interface
DDPG	Deep deterministic policy gradient
ROS	Robot operating system
v	Linear velocity
w	Angular velocity
x	Longitude signal information
y	Latitude signal information
h	heading angle signal information
A	System state matrix
B	System input matrix
C	System output matrix
K	Controller gain
